# Covalent vs. Dative Bonding in Carbon Monoxide and Other 10-Valence-Electron Diatomics

**DOI:** 10.3390/molecules29225396

**Published:** 2024-11-15

**Authors:** Khadija Rizwan, John Morrison Galbraith

**Affiliations:** Department of Chemistry, Biochemistry and Physics, Marist College, 3399 North Road, Poughkeepsie, NY 12601, USA; khadija.rizwan1@marist.edu

**Keywords:** valence bond theory, dative bonds, charge-shift bonding, resonance energy

## Abstract

Valence bond theory (VB) was used to determine the extent and driving forces for covalent vs. dative bonding in 10-valence-electron diatomic molecules N_2_, CO, NO^+^, CN^−^, P_2_, SiS, PS^+^, and SiP^−^. VBSCF calculations were performed at the CCSD(T)/cc-pVDZ optimized geometries. The full triply bonded system included 20 VB structures. A separation of the σ and π space allowed for a subdivision of the full 20 structure set into sets of 8 and 3 for the π and σ systems, respectively. The smaller structure sets allowed for a more focused look at each type of bond. In situ bond energies for σ bonds, individual π bonds, the π system, and triple bonds follow expected trends. Our data shows that N_2_ and P_2_ have three covalent bonds whereas CO and SiS contain two covalent and one dative bond, and charged species NO^+^, CN^−^, PS^+^, and SiP^−^ are a mixture of N_2_ and CO type electronic arrangements, resulting in a nearly equal charge distribution. Dative bonds prefer to be in the π position due to enhanced σ covalency and π resonance. Both σ and π resonance energies depend on a balance of ionic strength, orbital compactness, σ constraints, and bond directionality. Resonance energy is a major contributor to bond strength, making up more than 50% of the π bonds in SiS and PS^+^ (charge-shift bonds), and is greater than charge transfer in dative bonds.

## 1. Introduction

Carbon monoxide (CO) is a natural atmospheric pollutant and a chemical by-product of industrialization, and is present in blood at varying levels. For example, rural nonsmokers may have levels between 0.5 and 2%, while heavy smokers may have levels up to 9% [1]. However, when levels of carbon monoxide become elevated, it can be toxic. This is because CO binds to hemoglobin with a 220% greater affinity than oxygen [2], forming carboxyhemoglobin, which impairs the ability of blood to transport oxygen to the body’s organs and tissues. Oxygen binds to heme with the O_2_ axis at an angle, a binding conformation readily accommodated by myoglobin. In contrast, when CO binds to free heme, the Fe, C, and O atoms are strictly perpendicular to the heme plane, resulting in stronger binding due to less steric hindrance [3] and better backbonding from the Fe into the π* orbital of CO [4,5]. Coma or death can result if over half of the hemoglobin sites are occupied by CO molecules [6].

**Figure 1:**
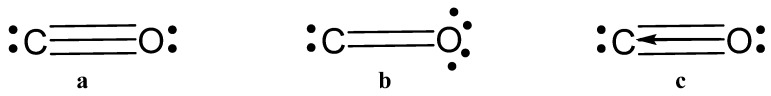
Lewis-type structural representations of carbon monoxide. In (**a**) octet rule is followed but negative formal charge resides on the more electronegative O atom. Structure (**b**) reduces formal charge but violate octet rule. Structure (**c**) uses arrow notation to indicate a dative bond.

For such a seemingly simple diatomic molecule, the bonding in CO is deceptively complex, as described by Frenking et al. [7,8]. Following the rules for drawing Lewis structures results in structure **1a** in Figure 1. While this structure follows the octet rule, it puts formal charge on the more electronegative O atom. Lewis structure **1b** in Figure 1 violates the octet rule on C but negates formal charge, which has been shown to override octet considerations in certain cases [9]. Experimentally, the bond length of CO is 1.1283 Å [10], in between that of triply bonded HCO^+^ (1.1047 Å [11]) and doubly bonded formaldehyde, H_2_CO, (1.205 Å [12]), indicating a combination of Lewis structures **1a** and **1b**, with structure **1a** being dominant. This is in agreement with the slight dipole moment (0.11D [13]) with the negative end on the C.

An alternative representation using arrows [14] is shown in Figure 1c. Though this is not without controversy [15,16], the arrow notation indicates the presence of a dative bond, a covalent bond where one species contributes both electrons to the two electron bond rather each species contributing a single electron as in a traditional covalent bond. In this case, the four valence electrons on C can form a lone pair, with two electrons free for bonding and an empty orbital, while the six valence electrons on O can form two lone pairs, with two electrons free for bonding. Pairing the free electrons on C and O results in two covalent bonds as in structure **1b**, and allowing one of the lone pairs on O to donate into the empty orbital on C forms a dative bond.

Structure **1c** is a convenient way of combining structures **1a** and **1b** into one compact representation that preserves the octet of each atom while minimizing formal charge. However, it raises a few interesting questions. Is the dative bond 100% dative, or is there some pure covalent contribution? Is the dative bond in the σ or π position? How much charge transfer is involved in the dative bond?

While Frenking and co-workers [7] analyzed bonding conundrums in CO using molecular orbital theory (MO) theory, electron density topology [17], and an energy decomposition analysis [18], herein we seek to use valence bond theory (VB) to specifically address the dative vs. covalent nature of the CO bonding system. As a superposition of states (Figure 1), VB is well situated to determine the contribution of covalent vs. dative type structures. VB also provides a clear and concise way to gauge the energy of a bond in situ [19,20,21], without disturbing the molecular environment. Lastly, VB has been instrumental in uncovering a new class of chemical bonding, charge-shift bonding (CSB) [22], where structure mixing resulting in a large resonance energy stabilization is the driving force of the bond.

In order to gain a more complete understanding of the unique bonding situation in CO, we will start with the isoelectronic N_2_ molecule. The knowledge gained in this regard will serve as a base from which we can analyze CO and other diatomic molecules with 10 valence electrons such as CN^−^, NO^+^, P_2_, PS^+^, and SiS.

## 2. Results and Discussion

Table 1 contains the components of the σ and π covalent and dative bonds for N_2_, CO, NO^+^, CN^−^, P_2_, SiS, PS^+^, and SiP^−^. Dative bond components for N_2_ and P_2_ cannot be measured because their neutral states are covalent in nature. We note that our values for total bond energies, *BE_T_*, are significantly above experimental bond dissociation energies [23]. However, in our values, the Pauli repulsion and electrostatic interactions, which Frenking and co-workers [7,24] found to be of major importance, are present both in the non-bonded reference state and the bound state, and therefore cancel out. While our *BE* values are more closely aligned with Frenking’s Δ*E*_int_ values, they were calculated at the VBSCF level, and therefore lack dynamic correlation in the bonds resulting in considerably lower values. 

In addition, we note that the sum of the σ and π bond energies is consistently lower than the total bond energies for all molecules herein. This is not a matter for concern considering that the calculations of individual σ and π bond energies are limited in the VB active space (3 and 8 VB structures for the σ and π systems, respectively) whereas the total bond contains VB structures that mix σ and π systems (20 in total). For similar reasons, the *BE* of single π bonds are less than half the *BE* of the full π system. As a result, we are careful not to make direct comparisons between the results reported herein and previous experimental and calculated results, but rather look for qualitative trends.

For all molecules in Table 1, the σ bond is stronger than a single π bond, as expected. Conversely, the π bonds have greater *RE_CS_* than the σ bonds. The greater π *RE_CS_* has been attributed to a weakening of the π bond due to the constraints of the σ bond preventing optimal *p* orbital overlap [20]. In the case of dative bonds (Table 1, columns 7 and 8) *RE_CT_* is greater for a σ than a π dative bond in all cases except CN^−^ and SiP^−^ where they are nearly identical. In the case of *RE_CT_*, there are two competing effects: σ constraints favor higher resonance energies for π than σ bonds as with *RE_CS_*, but the directionality of the σ orbitals favors mixing in the covalent type structure, leading to higher σ *RE_CT_*. For CN^−^ and SiP^−^, the π weakening effect dominates due to the extra constraint imposed by the σ bond, as can be seen by the bond lengths that are noticeably longer than other members of their group. We also note the sizable *RE_π_* values in column 8 due to including both π_x_ and π_y_ dative VB structures, indicating that resonance among dative structures is more significant than charge transfer.

For σ covalent bonds, resonance energies increase going from N_2_ to CO and NO^+^ and then decrease for CN^−^ (with a similar trend for third-row analogs). Resonance energies depend on two factors, the stabilization of ionic structures, which correlates with the difference in electronegativity values, and ionic structures serving to lower the kinetic energy to obtain the viral ratio [25,26,27], which depends on the compactness of orbitals and correlates with the sum of electronegativity values [20]. The *RE_CS_* of CO is greater than that on N_2_ due to the high electronegativity of O stabilizing the C^+^ :O^−^ ionic structure. There is little energetic incentive for the N^+^O electronic arrangement in the covalent VB structure or to shift electrons onto the less electronegative N atom or to adopt a N^2+^ O^−^ electron arrangement [28]. However, the compact orbitals of both N and O are made even more so by the positive charge, leading to a higher *RE_CS_* than CO. Lastly, CN^−^ suffers from both high energy ionic structures and diffuse orbitals, resulting in the lowest *RE_CS_* value. This argument also holds for third-row analogs. The situation is slightly different for π covalent bonds with a smaller *RE_CS_* for NO^+^ and PS^+^ than CO and SiS, respectively. In this case, the side-by-side arrangement of *p* orbitals leads to smaller contribution from orbital contraction effects, and the more stable C^+^O^−^ and Si^+^S^−^ structures dominate.

**Figure 2:**
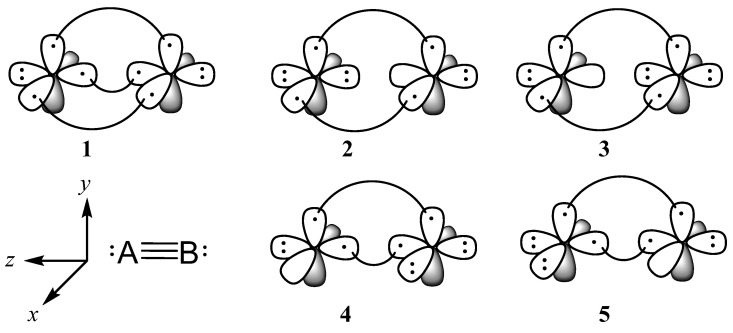
A subset of the 20 structures used in the present work. For mixed diatomics, B is the more electronegative atom. Structure **1** is the covalent (spin-paired) structure for both σ and π bonds, structures **2** and **3** are the σ ionic structures, and structures **4** and **5** are the ionic structures for the π*_x_* bond. The π*_y_* bond has structures similar to **4** and **5** in the *zy*-plane.

For N_2_, the spin-paired all-covalent VB structure (Figure 2, structure **1**) dominates with a weight of 0.351 (Table 2). The σ ionic structures (Figure 2 structures **2** and **3**) have the next highest weight (0.069), with π ionic structures even lower (0.051). This leads to a picture of bonding in N_2_ that is covalent in nature with little contribution from ionic structures in both the σ and π space, well below the 50% marker for charge shift bonds [22].

On the other hand, in the full 20-structure VBSCF calculation of CO, the highest weighted structures (0.259, Table 2) place the dative bond in the π system (Figure 2, structure **4** and the analogous counterpart with the dative bond in the *zy*-plane), followed by placing the dative bond in the σ position (0.169) and the all-covalent structure (0.135). All other structures contribute less than 0.100. These values indicate that CO is triply bound with one dative and two covalent bonds rather than three covalent bonds and that the dative bond prefers to be in the π position. The positional preference of the dative bond is also borne out by the energies of individual structures (Table 2); placing the dative bond in the π position is 214.6 and 408.0 kJ/mol lower in energy than the all-covalent and σ dative bond structures, respectively.

**Figure 3:**
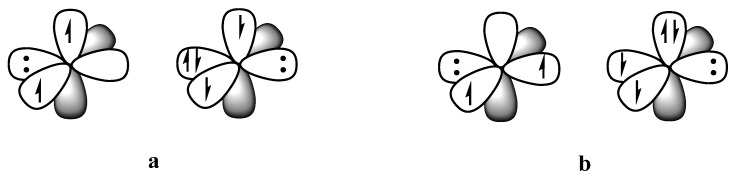
Non-bonded determinants with dative electrons in the σ (**a**) and π (**b**) positions.

**Figure 4:**
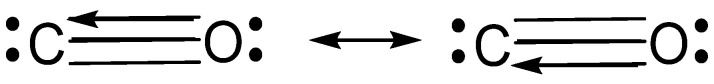
Lewis-type structural representation of CO indicating the resonance of dative bonds in the π position.

We can break this ordering down further by taking a deeper look into each individual interaction. When turning all other bonding interactions off by considering single determinants, placing the two dative electrons in the σ position (Figure 3**a**) is energetically favorable by 67.8 kJ/mol [30]. This is reasonable considering the greater stabilization of the dative electrons in the σ position pointing directly at the C center rather than perpendicular when in the π position. However, when bonding interactions are turned on, the benefits of the stronger σ covalent bond outweigh the benefits of having the dative bond in the σ position, resulting in the energetic preference for structure **4** over **3** (Table 2). Furthermore, there is a large resonance stabilization gained by having two possible positions for the dative bond in the π system. Overall, the picture can be represented as in Figure 4, with resonance between structures with the dative bond in the π*_x_* and π*_y_* positions.

Esterhuysen and Frenking attribute the greater bond strength of CO over N_2_ to the much reduced Pauli repulsion in CO rather than resonance energy [24]. While we are careful not to make direct comparisons to Esterhuysen and Frenking’s results for the reasons detailed above, herein we find that the resonance energy makes a substantial contribution to σ and π bond energies of both CO and N_2_ but is greater for CO (Table 1). We also note that the covalent contribution is also greater for CO than N_2_.

The best description of NO^+^, and CN^−^ depends on the location of the charge. In NO^+^, for example, if an electron is removed from the N, the bonding situation is similar to CO with one dative and two covalent bonds. However, if the O carries the positive charge, it becomes isovalent with N, leading to a bonding situation similar to the triple bond in N_2_. Similarly, for CN^−^, placing the negative charge on C results in a bonding situation similar to N_2_, whereas placing it on N is similar to CO.

The experimental ionization energies are 13.61 and 14.53 eV for O and N, respectively [31], while the electron affinities are 1.26 and −0.07 eV for C [32] and N [33], respectively, indicating a preference for NO^+^ and C^−^N type structures. However, the Mulliken charges of N^0.461^O^0.539^ and C^−0.573^N^−0.427^ indicate a nearly equal contribution from the N^+^O and CN^−^ dative type structures. While Table 2 has the covalent structure making the highest contribution for NO^+^ and CN^−^, the dative type structures are not insignificant, especially when considering that there are two structures of type **4** and that structures **3** and **4** (and its *y* counterpart) both lead to N^+^O and CN^−^ charge distributions. 

Overall, NO^+^ and CN^−^ are triply bonded with a nearly even mix of all-covalent and two covalent/one dative bond electron distributions. As with CO, the dative bond prefers to be in the π position. In the absence of other bonding interactions, the dative bond prefers to be in the σ position due to increased overlap by 196.8 and 230.6 kJ/mol for NO^+^ and CN^−^, respectively. However, when spin pairing is turned on, placing the dative bond in the π position allows for a more stable σ spin pairing as well as π resonance between two different π positions, leading to lower energies for VB structures with the dative bond in the π rather than σ position (Table 2).

Third-row analogs of second-row molecules suffer from poor atomic orbital overlap and weaker hybridization, leading to numerous effects such as trans bending rather than planar π bonding [21]. The poor overlap is apparent in the weaker bonding of both σ and π components of third-row molecules (Table 1). As a result, the third-row analogs P_2_, SiS, PS^+^, and SiP^−^ have longer bond lengths and weaker triple bond energies (Table 1). This decrease in bond energy is driven by the σ bonds, which decrease by an average of 383.5 kJ/mol compared to 161.2 kJ/mol for the π bonds. Resonance energies also decrease upon moving to the third row, although at a slower rate, leading to the resonance energies making up a larger percentage of the bond energy, increasing the charge-shift character. For SiS and PS^+^, *RE_CS_* makes up more than 50% of the π bond, putting them in the class of charge-shift bonds [22].

SiS, PS^+^, and SiP^−^ follow the same trends as CO, NO^+^, and CN^−^, with the dative bonds preferring to be in the π position. In the absence of other bonding interactions, the determinant with the dative electrons in the σ position is more stable by 9.4, 105.8, and 129.1 kJ/mol for SiS, PS^+^, and SiP^−^, respectively. However, allowing for spin pairing puts structure **4** below **3** due to better σ covalent bonding and π resonance (Table 2). Experimental ionization energies [31] of P (10.49 eV) and S (10.36 eV) indicate a slight preference for the PS^+^ covalent arrangement, but the Mulliken charges of P^0.525^S^0.475^ indicate a strong contribution from the P^+^S dative arrangement. Likewise, experimental electron affinity values for Si (1.39 eV) [34] and P (0.746 eV) [35] favor a Si^−^P covalent arrangement but the Mulliken charges of Si^−0.505^P^−0.495^ indicate a strong contribution from the SiP^−^ dative arrangement.

## 3. Theoretical Methods

**Figure 5:**
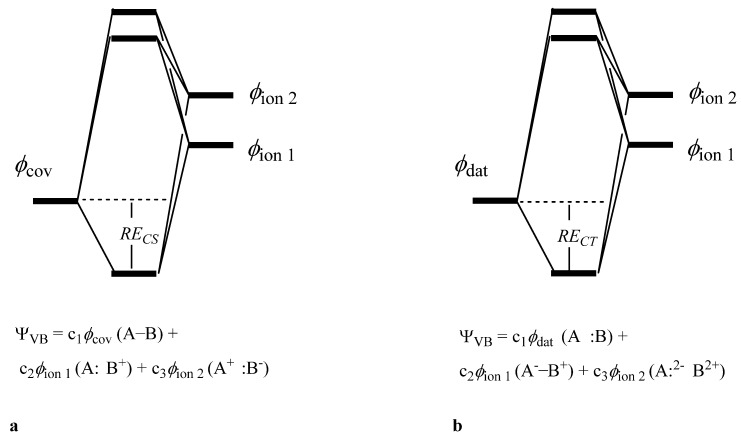
Valence bond descriptions of (**a**) covalent and (**b**) dative bonds. In the covalent case (**a**), the spin-paired state, *f*_cov_(A−B), is low in energy and electronically neutral. In this case, A is more electronegative than B, resulting in *ϕ_ion 1_* (A:^−^ B^+^) being lower in energy than *ϕ_ion 2_* (A^+^ :B^−^). In the dative case (**b**), species B donates both electrons to the bond, resulting in *ϕ_dat_* being the low energy/electronically neutral structure while the spin-paired structure, *ϕ_ion 1_*, carries charge.

A two-electron bond can be described with the 3 structures in Figure 5 [36]. However, the situation is more complicated for a triply bonded system such as N_2_ and its analogs, where the structures describing each bond can mix with the other bonds, resulting in 175 structures. The complete wavefunction is a superposition of all these structures as in Equation (1), where *ϕ*_K_ are VB structures and *C_K_* are structural coefficients.

(1) $$\psi_{VB}=\sum_{K}C_{K}\phi_{K}$$

In addition to being cumbersome to work with, not all structures make a meaningful contribution to the overall wavefunction, so we used a truncated set of the 20 highest contributing and most chemically meaningful structures, as seen in Figure S1 of Supplemental Information. Of these 20, the 5 used for illustrative purposes herein are displayed in Figure 2. 

While the complete description of a 6 electron/6 orbital system requires 5 spin-paired Rumer structures [37], we separated the σ and π systems to the extent possible, resulting in a 4-electron/4-orbital π system with 2 spin-paired structures (**1** and **8**, Figure 2 and Figure S1) and a 2-electron/2-orbital σ system requiring a single spin-paired structure. Further truncation of this set of 20 VB structures will be described where appropriate below. 

**Figure 6:**
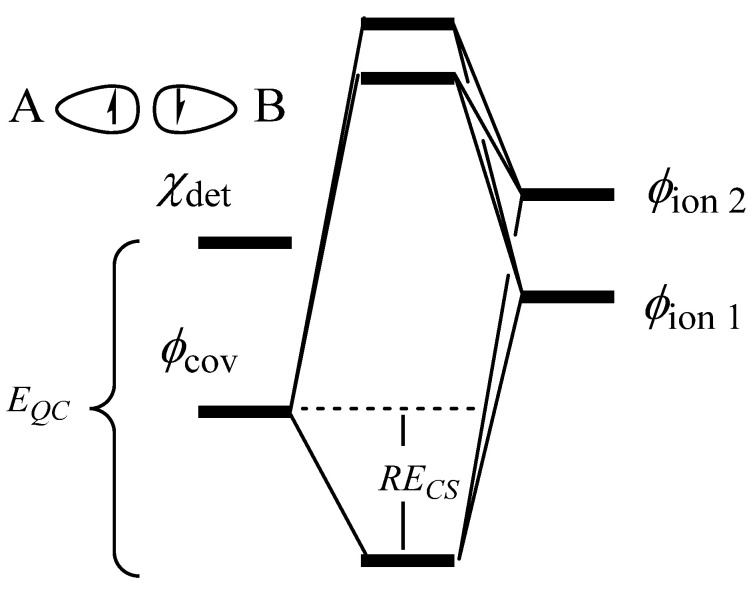
Quasiclassical determination of in situ energy of a 2-electron bond.

The spin structure *ϕ*_cov_ in Figure 5a is made up of 2 interacting determinants of opposite spin, A(*α*) (*β*)B and A(*β*) (*α*)B. Considering just one of these determinants, *χ_det_* in Figure 6 is equivalent to turning off the bonding interaction. The difference between this non-bonded reference and all three structures is then a measure of the quasiclassical bond energy, *E_QC_* [19,20,21]. The quasiclassical energy is a measure of the bond energy in situ, within the framework of the molecule. There is still a question of how to treat the remaining bonds in the system. This question is fairly straight forward for N_2_ where each N has 3 unpaired electrons that can spin-pair to form 3 covalent bonds. In this case, the non-bonded reference confines the bond in question to a single spin configuration, *αβ* or *βα*, while the other bonds are held in their covalent VB arrangement, which allows both possible spin arrangements, *αβ* and *βα*. 

However, in CO, the O atom has 2 unpaired electrons and 2 lone pairs while the C atom can be thought of as having 2 unpaired electrons and 1 lone pair. Given this arrangement, the electronically neutral state involves 2 spin-paired bonds and 1 dative bond. It is possible to consider a covalent or dative bond in one position by forcing the other type of electronic arrangement in the other position. Thus, the non-bonded reference for a π covalent bond confines one π bond to a single spin determinant, allows the other π bond to have both spin determinants, and places the 2 σ electrons in the dative electronic arrangement. A similar approach has been taken for NO^+^, CN^−^, SiS, PS^+^, and SiP^−^.

When considering multiple bonds at the same time, for example, both bonds of the π system, there are two possible arrangements of spin determinants, π_x_(*αβ*) π_y_(*αβ*) and π_x_(*αβ*) π_y_(*βα*), one of which will be stabilized by exchange, π_x_(*αβ*) π_y_(*αβ*). In this case, we follow the procedure of Shaik et al. [21] and take the average energy as the non-bonded reference. Similarly, when considering both π bonds and the σ bond, we take the average of three spin determinants: π_x_(*αβ*) π_y_(*αβ*) σ(*αβ*)*,* π_x_(*αβ*) π_y_(*αβ*) σ(*βα*), and π_x_(*αβ*) π_y_(*βα*) σ(*αβ*). This amounts to including exchange for all bonds, between the π bonds, and between one π and the σ bond.

With the non-bonded reference defined, we determine the energy of one covalent bond, either π or σ, as the difference between the non-bonded reference and the 3 structures of Figure 5a for that bond only, while the other bonds are held in the same arrangement as in the non-bonded reference. The bond energy of the whole π system is determined as the difference between the non-bonded reference (average of 2 determinants in this case) and all 8 VB structures that make up the π system while keeping the σ system unchanged. The 8 structures included are the spin-paired structures **1** and **8**, the ionic structures **4**, **5**, **6,** and **7**, and the neutral charge transfer structures **9** and **10** (Figure 2 and Figure S1) [38]. Similarly, the bond energy of the entire triple bond is taken as the difference between the non-bonded reference state (average of 3 determinants) and the calculation including all 20 VB structures. Herein, quasiclassical bond energies are designated *BE*. Determining the bond energy of a dative bond in the same manner is not possible because the previously defined non-bonded reference state is charged (C^−^O^+^) but the neutral dative spin arrangement with both electrons on the O has some stabilization due to the C nuclear center. Rather, for dative bonds, we focus on the charge transfer and π resonance energy as defined below. By convention, we denote molecule AB with B as the more electronegative atom. When discussing charge transfer in dative bonds, we are always referring to transfer from B to A.

VB theory allows for the possibility of further dividing up the total bond energy into individual contributions. For σ bonds, the covalent contribution, *E_cov_*, is the difference between the non-bonded reference and the covalent VB structure alone. A similar approach is taken for an isolated π bond, π_1_. However, considering both π bonds together results in a 4-electron/4-orbital system requiring 2 spin-paired structures. In this case, the covalent contribution of the π system, π_2_, is taken as the difference between the non-bonded reference and covalent structures **1** and **8** (Figure 2 and Figure S1) [39]. 

The resonance energy is then the difference between the covalent structure and the calculation including all three VB structures for that bond, as in Figure 5a. Bonds where the resonance energy is the main contributing factor are known as charge-shift bonds [22], and therefore this resonance energy is referred to as *RE_CS_* herein. In the case of a dative bond, the difference between the dative structure alone and all three VB structures gives a measure of the charge transfer contribution and is designated *RE_CT_*. We define *RE_CT_* for the full π system, π_2_, as the difference between the 2-structure VB calculation with the dative electrons in the *x* and *y* positions and the full 8-π structure VB calculation.

The principle of resonance applies to any situation where there is a stabilization due to mixing VB structures. In this framework, we define the dative π resonance energy as the stabilization due to the possibility of having the dative electrons in two possible arrangements, *x* or *y*, in Figure 2. This stabilization, designated *RE_π_*, is the energetic difference between a single structure of type **4** in Figure 2, and a two-structure calculation including **4** and its corollary in the *zy* plane. The resonance energy of the π system, π_2_, is then the difference between the two 4-electron/4-orbital covalent structures, and the full 8-structure π active space. 

Weights of VB structures were determined by the method of Chirgwin and Coulson [29] (Equation (2)). Chirgwin–Coulson weights are defined as the square of the structural coefficient plus ½ the overlap with all other structures and are the VB analog of Mulliken populations in molecular orbital (MO) calculations. While there are other ways of determining structural weights, they generally give the same qualitative trends [36].

(2) $$W_{K}=\sum_{L}C_{K}C_{L}\langle \phi_{K}|\phi_{L}\rangle$$

The geometries of all molecules were optimized at the CCSD(T) [40,41,42,43]/cc-pVDZ [44] level of theory with Gaussian16 [45]. Valence bond theory calculations were performed using the Xiamen ab initio Valence Bond program (XMVB 3.0) [46,47], with the Valence Bond Self-Consistent Field (VBSCF) method [48,49] in conjunction with the cc-pVDZ basis set. In the VBSCF method, all VB structures share the same set of orbitals, which are optimized along with the structural coefficients (Equation (1)). While VBSCF includes static electron correlation, the inclusion of dynamic correlation requires a more rigorous method such as breathing orbital VB (BOVB) [50] that allows for a separate set of orbitals for each VB structure. However, in the present study, the large structure set made BOVB impractical. While BOVB leads to more accurate results, general trends and conclusions obtained with VBSCF have been shown to be the same as with BOVB [20].

## 4. Conclusions

Our results show that of the triply bonded diatomic molecules with 10 valence electrons, N_2_ and P_2_ have three covalent bonds whereas CO and SiS contain two covalent and one dative bond. Charged species NO^+^, CN^−^, PS^+^, and SiP^−^ are a nearly even split of these two bonding types which serves to distribute the charge. When a dative bond is present, it prefers to be in the π position, thereby allowing for the stronger σ covalent bond and stabilization due to resonance among structures with the π bond in the *x* and *y* planes, *RE_π_*. In π dative bonds, stabilization from *RE_π_* is greater than from charge transfer.

Constraints imposed by the σ bonds enforce a non-optimal situation for π bonding, leading to weakened bonds and resonance energies that are greater than σ resonance energies. The values of resonance energies depend on a balance between the strength of ionic structures, orbital compactness, σ constraints, and bond directionality, resulting in the SiS and PS^+^ π bonds falling into the category of charge-shift bonds, with more than 50% of the bond energy coming from resonance stabilization. In all cases, the resonance energy (both σ and π) makes a significant contribution to the overall bond energy.

## Figures and Tables

**Table 1:** Breakdown of bond energies into resonance energies and covalent contributions. Bond energies in kJ/mol and bond lengths in Å. N_2_ and P_2_ are predominantly covalent in nature and do not have *RE_CT_* or *RE_π_* values as indicated by *.

		*E_cov_*	*RE_CS_*	*BE*	%*RE_CS_*	*RE_CT_*	*RE*_π_	*r_e_*	*BE_T_*
N_2_	σ	656.4	97.9	754.3	12.9	*		1.119	1759.0
	π_1_	246.6	159.8	406.3	34.7	*	*		
	π_2_	542.1	378.0	920.1	41.1	*			
CO	σ	736.0	134.5	870.5	15.4	395.3		1.145	2270.3
	π_1_	251.2	325.5	576.7	56.4	230.5	399.9		
	π_2_	543.6	595.0	1138.6	52.2	132.5			
NO^+^	σ	867.8	167.7	1035.5	16.2	851.0		1.079	2328.8
	π_1_	253.0	237.2	490.2	48.4	554.5	446.0		
	π_2_	561.6	529.9	1091.4	48.6	441.1			
CN^−^	σ	530.3	57.4	587.7	9.8	430.9		1.200	1439.2
	π_1_	228.2	119.5	347.8	34.6	435.0	334.1		
	π_2_	485	283.6	769.2	36.9	281.0			
P_2_	σ	281.2	51.9	333.0	15.5	*		1.937	757.7
	π_1_	77.8	64.4	142.2	45.3	*	*		
	π_2_	209.9	149.5	359.4	41.6	*			
SiS	σ	331.0	80.5	411.5	19.6	146.9		1.972	1136.4
	π_1_	89.3	191.2	280.6	68.2	77.9	174.9		
	π_2_	222.4	309.2	531.6	58.2	45.5			
PS^+^	σ	383.1	82.7	465.7	17.8	366.3		1.872	1054.6
	π_1_	90.3	103.5	193	53.4	238.8	194.2		
	π_2_	234.8	222.3	457.1	48.6	196.1			
SiP^−^	σ	245.4	34.6	279	12.4	190.6		2.053	653.3
	π_1_	76.8	49.2	126.0	39.0	195.9	142.3		
	π_2_	190.8	114.2	305.1	37.4	139.1			

**Table 2:** Structural weights and energies for VB structures **1**, **3**, and **4** from Figure 2. Weights from full 20-structure VB calculations according to the method of Chirgwin and Coulson [29] (Equation (2)). Energies for individual structures in kJ/mol relative to lowest energy structure.

		1	3	4
N_2_	*W_CC_*	0.351	0.069	0.051
	*E*	0.0	822.1	748.4
CO	*W_CC_*	0.135	0.169	0.259
	*E*	214.6	408.0	0.0
NO^+^	*W_CC_*	0.254	0.135	0.154
	*E*	0.0	647.2	234.1
CN^−^	*W_CC_*	0.329	0.129	0.124
	*E*	0.0	359.2	295.4
P_2_	*W_CC_*	0.411	0.071	0.042
	*E*	0.0	419.5	414.7
SiS	*W_CC_*	0.090	0.176	0.297
	*E*	222.8	255.8	0.0
PS^+^	*W_CC_*	0.260	0.146	0.169
	*E*	0.0	254.2	72.5
SiP^−^	*W_CC_*	0.344	0.152	0.137
	*E*	0.0	156.1	124.0

## Data Availability

The original contributions presented in this study are included in the article/Supplementary Materials. Further inquiries can be directed to the corresponding author.

## Supplementary Materials

The following supporting information can be downloaded at: https://www.mdpi.com/article/10.3390/molecules29225396/s1, Figure S1: Full structure set for VBSCF calculations.; Table S1: Chirgwin-Coulson weights of VB structures. Structure numbering refers to Figure S1.
