# Supplementary material for: Covalent vs. Dative Bonding in Carbon Monoxide and Other 10-Valence-Electron Diatomics
Source: Molecules. 2024 Nov 15;29(22):5396. doi: 10.3390/molecules29225396 (PMC11596940; doi:10.3390/molecules29225396)
Supplement: Supplementary file 1 [file molecules-29-05396-s001.zip › molecules-3304826-supplementary.pdf]

Supplemental information for

Covalent vs. Dative Bonding in Carbon Monoxide and Other 10-Valence-Electron Diatomics

Khadija Rizwan and John Morrison Galbraith

Department of Chemistry, Biochemistry and Physics, Marist College, 3399 North Road, Poughkeepsie, NY 12601, USA

Table S1. Chirgwin-Coulson weights of VB structures. Structure numbering refers to Figure S1.

	N_2	CO	NO^+	CN^-	P_2	SiS	PS^+	SiP^-
**1**	0.351	0.135	0.254	0.329	0.411	0.090	0.260	0.344
**2**	0.069	0.005	0.027	0.007	0.071	0.002	0.020	0.002
**3**	0.069	0.170	0.135	0.129	0.071	0.175	0.146	0.152
**4**	0.051	0.259	0.154	0.124	0.042	0.2976	0.169	0.137
**5**	0.051	-0.003	0.009	0.001	0.042	-0.003	0.003	-0.006
**6**	0.051	-0.003	0.009	0.001	0.042	-0.003	0.003	-0.006
**7**	0.051	0.259	0.154	0.124	0.042	0.2976	0.169	0.137
**8**	-0.028	-0.017	-0.022	-0.028	-0.041	-0.018	-0.028	-0.038
**9**	0.049	0.013	0.033	0.046	0.044	0.003	0.027	0.035
**10**	0.049	0.013	0.033	0.046	0.044	0.003	0.027	0.035
**11**	0.048	0.019	0.046	0.046	0.049	0.010	0.048	0.050
**12**	0.048	0.007	0.023	0.039	0.049	0.001	0.018	0.030
**13**	0.048	0.019	0.046	0.046	0.049	0.010	0.048	0.050
**14**	0.048	0.007	0.023	0.039	0.049	0.001	0.018	0.030
**15**	0.004	-0.001	-0.000	-0.002	0.002	-0.000	-0.001	-0.002
**16**	0.010	0.000	0.003	-0.001	0.007	0.000	0.002	-0.002
**17**	0.010	0.000	0.003	-0.001	0.007	0.000	0.002	-0.002
**18**	0.010	0.030	0.021	0.020	0.007	0.028	0.020	0.019
**19**	0.004	0.059	0.026	0.015	0.002	0.076	0.030	0.016
**20**	0.010	0.030	0.021	0.020	0.007	0.028	0.020	0.019

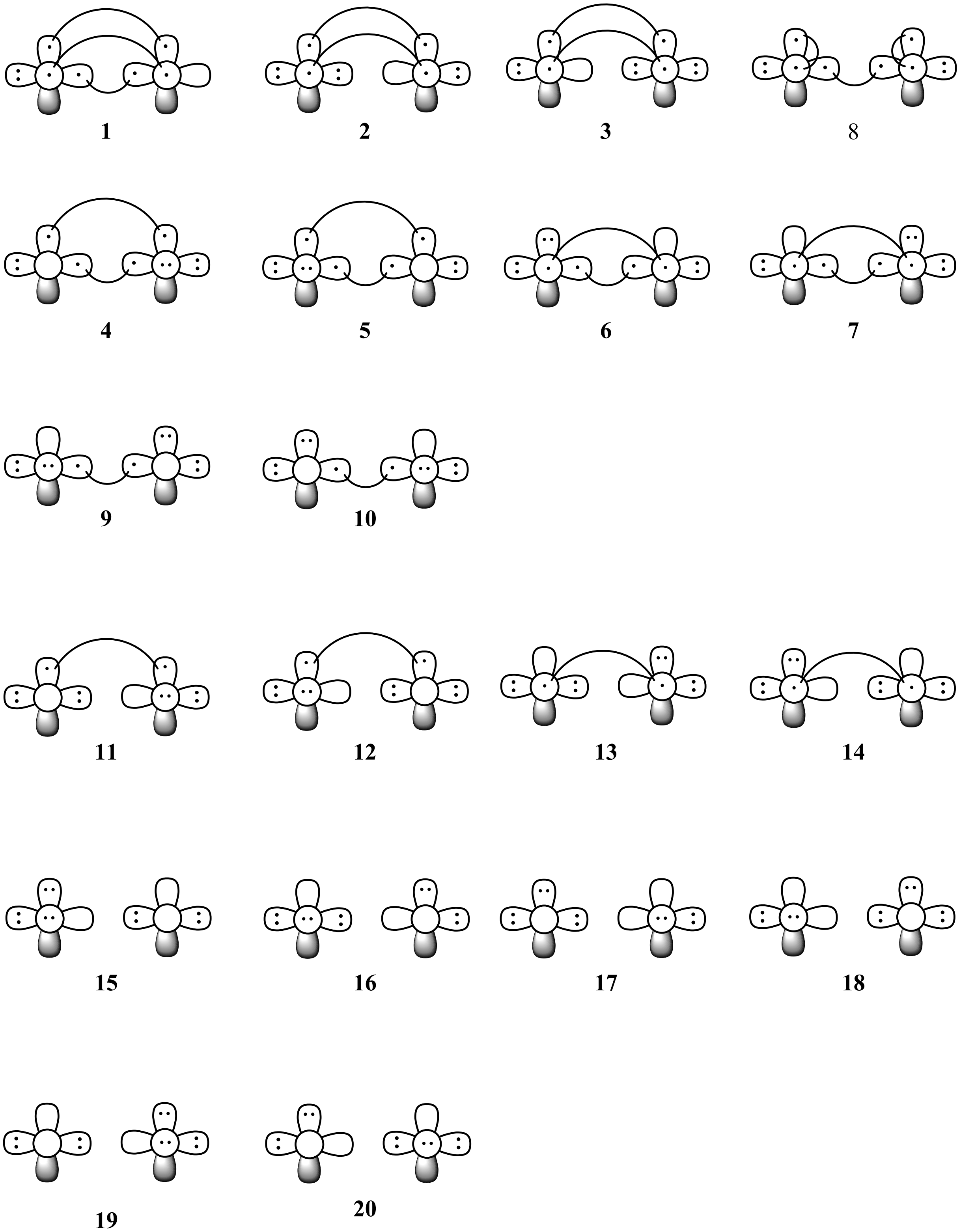

Figure S1. Full structure set for VBSCF calculations.
